# Accelerometer-based sedentary time and physical activity from childhood through young adulthood with progressive cardiac changes: a 13-year longitudinal study

**DOI:** 10.1093/eurjpc/zwae129

**Published:** 2024-05-07

**Authors:** Andrew O Agbaje

**Affiliations:** Clinical Epidemiology and Child Health Unit, Institute of Public Health and Clinical Nutrition, School of Medicine, Faculty of Health Sciences, University of Eastern Finland, Yliopistonranta 8, P.O. Box 1627, 70211 Kuopio, Finland; Children’s Health and Exercise Research Centre, Department of Public Health and Sports Sciences, Faculty of Health and Life Sciences, University of Exeter, Exeter, UK

**Keywords:** Movement behaviour, Left ventricular hypertrophy, Paediatrics, Health guidelines, Hypertension, Obesity, Health promotion

## Abstract

**Aims:**

Longitudinal evidence on the relationship of sedentary time (ST), light-intensity physical activity (LPA), and moderate-to-vigorous-intensity physical activity (MVPA) with changes in cardiac structure and function in the paediatric population is scarce. This evidence is clinically important due to the impact ST can have on the long-term prognosis of healthy young population in the lifetime continuum. This prospective observational study examined the relationships of cumulative ST, LPA, and MVPA from childhood with longitudinal changes in cardiac structure and function.

**Methods and results:**

This is a secondary analysis from the Avon Longitudinal Study of Parents and Children, UK birth cohort of 1682 children aged 11 years. Participants who had at least one follow-up timepoints accelerometer-measured ST, LPA, and MVPA over a period of 13 years and repeated echocardiography-measured cardiac structure and function at ages 17- and 24-year clinic visit were included. Left ventricular mass indexed for height^2.7^ (LVMI^2.7^) and left ventricular (LV) diastolic function from mitral E/A ratio (LVDF) were computed. Among 1682 children (mean [SD] age, 11.75 [0.24] years; 1054 [62.7%] females), the cumulative one-min/day increase in ST from ages 11 to 24 years was associated with progressively increased LVMI^2.7^ {effect estimate 0.002 g/m^2.7^ [confidence interval (CI) 0.001–0.003], *P* < 0.001}, irrespective of sex, obesity, and hypertensive status. Cumulative one-min/day increase in LPA was associated with a decreased LVMI^2.7^ (−0.005 g/m^2.7^ [−0.006 to −0.003], *P* < 0.0001) but an increased LVDF. Cumulative one-minute/day increase in MVPA was associated with progressively increased LVMI^2.7^ (0.003 g/m^2.7^ [0.001–0.006], *P* = 0.015).

**Conclusion:**

ST contributed +40% to the 7-year increase in cardiac mass, MVPA increased cardiac mass by +5%, but LPA reduced cardiac mass by −49%. Increased ST may have long-term pathologic effects on cardiac structure and function during growth from childhood through young adulthood; however, engaging in LPA may enhance cardiac health in the young population.

## Graphical Abstract

**Graphical Abstract:**
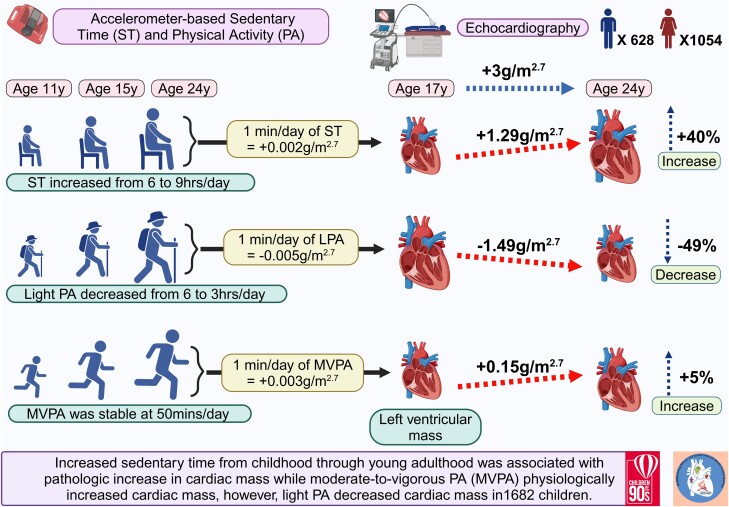

## Introduction

Among adults, higher left ventricular mass (LVM) is a strong predictor of cardiovascular mortality and regressive changes in LVM among adults have been associated with low rates of clinical events.^1,2^ In the paediatric population, due to the rarity of clinical events, alterations in cardiac structure and functional indices are surrogate measures for signs of early cardiac damage.^3–7^ Recently, elevated blood pressure, increasing lipids, and higher arterial stiffness in adolescence were shown to temporally precede premature cardiac damage in young adulthood.^6–8^ The primary prevention of premature cardiac damage is of public health importance for the promotion of life-long cardiovascular health and decreasing the global burden of cardiovascular diseases.^6–8^ The benefit of increasing physical activity (PA) on cardiometabolic and vascular health among children and adolescents has been established, and emerging longitudinal studies are supporting this evidence.^9–15^ This necessitates the recent recommendation of an average daily 60 min/day of moderate-to-vigorous-intensity PA (MVPA) in <18-year-olds but rarely do the paediatric population meet this daily threshold.^9,16^

Little is known about the independent longitudinal effect of modifiable lifestyle factors such as device-measured sedentary time (ST) and PA on changes in repeated echocardiography-measured cardiac structure and function in a general paediatric population.^9,15,17–21^ Moreover, the sex-specific relationship of ST and MVPA with cardiac structural and functional indices in the paediatric population remains unclear.^22^ It is also important to clarify these relationships in youth at risk of obesity and hypertension.^8,11,23,24^ A recent cross-sectional study of 530 adolescents concluded that ST was associated with a 30% higher cardiac mass, MVPA was associated with a 10% higher cardiac mass, and light-intensity PA (LPA) was associated with better cardiac function.^23^ Since children and adolescents accumulate more time engaging in LPA than MVPA, it is essential to clarify whether cumulative increases in LPA and MVPA independently associate with progressively better cardiac structure and function in youth.^17,23^ LPA from childhood was recently shown to be significantly more effective than MVPA in lowering fat mass, cholesterol levels, inflammation, and vascular stiffness and may be of public health importance.^11–15,25^

Due to the existing knowledge gaps, the following research questions remained unanswered: (i) what are the longitudinal relationships of each of ST, LPA, and MVPA with cardiac structural and functional indices? and (ii) do the relationships differ by sex, obesity, and hypertensive status? Thus, this present study examined the longitudinal associations of objectively and repeatedly measured ST, LPA, and MVPA from ages 11 to 24 years with cardiac structure and function in young adulthood (age 24 years) and changes in cardiac structure and function from ages 17 to 24 years (adolescence through young adulthood) using data from the Avon Longitudinal Study of Parents and Children (ALSPAC) birth cohort, England, UK.

## Methods

### Study cohort

**Figure 1:**
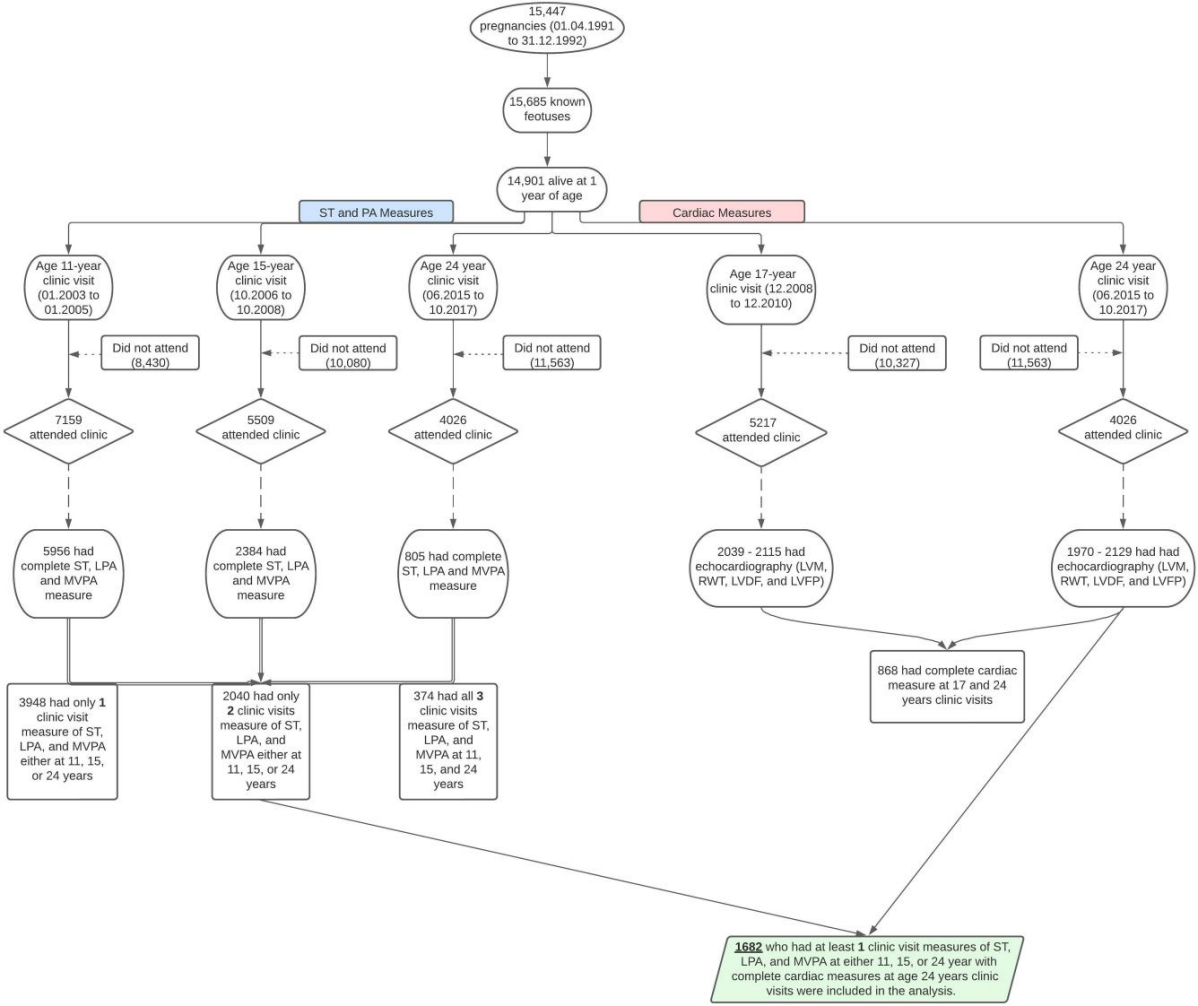
Flowchart of Avon Longitudinal Study of Parents and Children (ALSPAC) birth cohort participants. ST, sedentary time, LPA, light-intensity physical activity; MVPA, moderate-to-vigorous-intensity physical activity; LVDF, left ventricular diastolic function; LVFP, left ventricular filling pressure; LVM, left ventricular mass; RWT, relative wall thickness.

Details of the ALSPAC birth cohort have been published earlier.^26–29^ The ALSPAC birth cohort investigates factors that influence childhood development and growth. Altogether, 14 541 pregnancies from women residing in Avon, southwestern England, UK, who had a total of 14 676 foetuses, with an expected date of delivery between 1 April 1991 and 31 December 1992 were enrolled. When the oldest children were approximately 7 years of age, an attempt was made to bolster the initial sample with eligible cases who had failed to join the study originally resulting in 913 additional pregnancies. The total sample size for analyses using any data collected after 7 years of age was 15 454 pregnancies, resulting in 15 589 foetuses. Of these, 14 901 were alive at 1 year of age. Regular clinic visits of the children commenced at 7 years of age and are still ongoing. Study data at 24 years were collected and managed using REDCap electronic data capture tools.^30^ For this analysis, 1682 participants were included who had at least one timepoint measure of sedentary time (ST), light-intensity physical activity (LPA), moderate-to-vigorous-intensity physical activity (MVPA) during clinic visits at 11, 15, or 24 years and complete measure of LVM indexed for height^2.7^ (LVMI^2.7^), relative wall thickness (RWT), left ventricular diastolic function E/A ratio (LVDF), and left ventricular filling pressure E/eʹ ratio (LVFP) measurements (*Figure 1*).

Ethical approval for the study was obtained from the ALSPAC Ethics and Law Committee and the Local Research Ethics Committees. Consent for biological samples was collected in accordance with the Human Tissue Act (2004). Informed consent for the use of data collected via questionnaires and clinics was obtained from participants following the recommendations of the ALSPAC Ethics and Law Committee at the time.

### Movement behaviour measures

ST, LPA, and MVPA were assessed with MTI ActiGraph AM7164 2.2 (LLC, Fort Walton Beach, FL, USA) accelerometer worn for 7 days at 11- and 15-year clinic visits, whereas at 24 years movement behaviour was assessed using ActiGraph GT3X + accelerometer device worn for four consecutive days.^11–13^ Accelerometer was worn around the waist and detects acceleration and deceleration in a vertical plane as a combined function of movement frequency and intensity. There is a strong absolute agreement between the ActiGraph^TM^ models (intra-class correlation coefficient 0.99 [95% confidence interval (CI) = 0.98–0.99], thus making it acceptable to use different models within a study.^31^ The device was worn during waking hours in at least two week days and one weekend day. A valid day was defined as providing data for at least 10 h per day (excluding sequences of 10 or more min with consecutive zero counts), and children were only included in the analyses if they provided at least three valid days of recording. The devices capture movement in terms of acceleration as a combined function of frequency and intensity. Data were recorded as counts that result from summing post-filtered accelerometer values (raw data at 30 Hz) into 60-s epoch units. Data were processed using Kinesoft software, version 3.3.75 (Kinesoft), according to established protocol. Activity counts per minute (cpm) threshold validated in young people were used to calculate the amount of time spent: for MVPA, > 2296 cpm; for LPA, 100–2296 cpm; and for ST, 0 to − < 100 cpm at ages 11 and 15 years, but 2020 cpm for the 24-year assessment.^29,32,33^ The Evenson cutpoint used in stratifying activity threshold has shown the best overall performance across all intensity levels and was suggested as the most appropriate cutpoint for youth.^34,35^ The MVPA established cutpoints^9^ of ≥60 min/day, 40–<60 min/day, and <40 min/day as the reference category were computed at ages 11, 15, and 24 years, respectively. There are no established cutpoints for ST and LPA; therefore, categorical analyses were not presented.

### Cardiac structural and functional measures

At 17 years, echocardiography was performed according to the American Society of Echocardiography guidelines^36,37^ by one of two experienced echocardiographers using an HDI 5000 ultrasound machine (Phillips Healthcare, Amsterdam, The Netherlands) equipped with a P4-2 phased array ultrasound transducer. At 24 years, echocardiography was performed by two experienced echocardiographers using a Philips EPIQ 7G ultrasound system equipped with a X5-1 transducer. Philips Q-station was used for the M-mode, 2D, and Doppler echo analyses, while TomTec software was used for the 3D echo analyses.^23,38^ Measures of cardiac structure were LVMI^2.7^ and RWT computed from septal wall thickness, posterior wall thickness, and LV diastolic diameter as previously described.^7^ Measures of cardiac function were LVDF and LVFP. Pulsed Doppler examinations of transmitral flow were recorded from the apical four-chamber view. For LV measurements, the sample volume was positioned between the mitral annulus and the tips of the mitral leaflets with the position adjusted to maintain the sample volume at an angle as near parallel to transmitral flow as possible with the participant in passive end expiration. The peak flow velocity of the early (E) and atrial (A) waves was measured from the three consecutive cardiac cycles displaying the highest measurable velocity profiles. A similar measurement (eʹ) was conducted at the tricuspid valve. Tissue Doppler echocardiography was performed in the four-chamber view on the lateral, inferior, and septal LV walls to obtain myocardial wall velocities. Data were acquired with the beam parallel to the wall of interest and with optimal settings to ensure no over-gain of the low-velocity signals. A 5 mm sample volume was placed at the level of the mitral valve annulus, and a loop of 8–10 cardiac cycles was recorded. The reproducibility of echocardiographic examinations was assessed by recalling 30 participants and repeating their measurements. The intra-class correlation of repeated measurements ranged from 0.75 to 0.93 (intra-observer) and 0.78 to 0.93 (inter-observer).^39^

### Anthropometric, cardiometabolic, and lifestyle factors measures

Anthropometry (height and weight) of participants at ages 17 and 24 years was assessed by observing standard protocols, and body mass index (BMI) was computed as weight in kilograms per height in metres squared. BMI of ≥25 kg/m^2^ was categorized as overweight and obese at age 24-year clinic visit. Heart rate and systolic and diastolic blood pressure were measured at ages 17 and 24 years using an Omron 705-IT as previously detailed.^29,40,41^ Elevated and hypertensive systolic blood pressure was categorized as systolic blood pressure ≥130 mmHg.^42^ Fasting blood samples at ages 17 and 24 years were collected, spun, and frozen at −80°C and later assayed for lipids, glucose, insulin, and high-sensitivity C-reactive protein as detailed previously.^29,40,43,44^ Total fat mass and lean mass were assessed using a dual-energy X-ray absorptiometry scanner at 17- and 24-year clinic visits. Questionnaires to assess smoking behaviour were administered at 17- and 24-year clinic visits. Question related to smoking in the last 30 days was used as a marker of current smoking status. At 17-year clinic visit, participants were briefly asked about their personal and family (mother, father, and siblings) medical history such as a history of hypertension, diabetes, high cholesterol, and vascular disease. The participant’s mother’s socioeconomic status was grouped according to the 1991 British Office of Population and Census Statistics classification.^45^

### Statistical analysis

Cohort descriptive characteristics were summarized as means and standard deviation, medians and inter-quartile ranges, or frequencies and percentages. Sex differences were explored using independent *t*-tests, Mann–Whitney *U*-tests, or χ^2^ tests for normally distributed, skewed, or dichotomous variables, respectively. Multicategory variables were analysed using a one-way analysis of variance. Normality was assessed by histogram curve, quantile–quantile plot, and Kolmogorov–Smirnov tests. A logarithmic transformation of skewed covariates was conducted, and normality was confirmed prior to further analysis.

**Figure 2:**
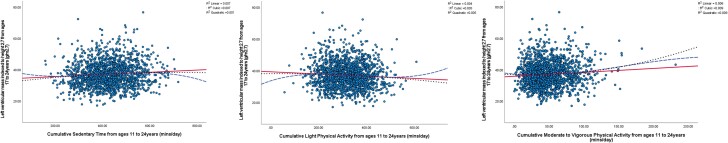
Unadjusted linear, cubic, and quadratic regression plots of cumulative sedentary time, light-intensity physical activity, and moderate-to-vigorous-intensity physical activity (min/day) from ages 11 to 24 years with progressive increase in left ventricular mass (g/m^2.7^) from ages 17 to 24 years. Linear regression is solid line, quadratic regression is short dash line, and cubic regression is long dash line.

The separate associations of the 13-year cumulative ST, LPA, and MVPA progression (ages 11–24 years) with each of the LVMI^2.7^, RWT, LVDF, and LVFP progressive changes repeatedly measured during ages 17- and 24-year follow-up were examined using generalized linear mixed-effect models (GLMMs) for repeated measures. The GLMM is robust for handling highly correlated variables such as ST and LPA with a Pearson correlation of −0.70. The optimal model was one with sex and predictor as a factor and a random intercept modelled on the subject level. Moreover, the best model fit was accepted for the model with the lowest Bayesian information criteria. A random effect variance component type was selected, and the effect of the predictor trajectory on the progressive change in outcome variables was determined. To test for fixed effects and coefficients, robust covariance estimation was selected to handle violations of model assumptions. A Little’s missing completely at random test was conducted to ascertain data missingness and concluded that the variables were not missing completely at random.^46^ While the GLMM is robust for handling missing at random predictor, covariate, and outcome data, an additional 20 cycles of multiple imputations were conducted to account for missing data predictor, covariates, and outcomes. Regression-modelled 20 cycles of multiple imputations were conducted using SPSS, version 27 (IBM Corp, Armonk, NY, USA), with 10 iterations generating 20 imputed data sets. The pooled imputed results after conducting multiple imputations and the GLMM were presented. The specified constraints for the imputation process were the observed minimum and maximum values, resulting in an estimate that was 98% efficient as computed with Rubin’s formula.^11,46^ The analysis strategy accounted for baseline ST, LPA, MVPA predictors, vascular outcomes, and covariates and their repeated measures. Model 1 was adjusted for sex, and other time-varying covariates measured at both baseline and follow-up such as age, low-density lipoprotein cholesterol, insulin, triglyceride, high-sensitivity C-reactive protein, high-density lipoprotein cholesterol, heart rate, systolic blood pressure, glucose, fat mass, lean mass, smoking status, family history of hypertension/diabetes/high cholesterol/vascular disease, and socioeconomic status. Model 2 was an additional mutual adjustment for ST, LPA, or MVPA depending on the predictor. Sex-based analyses were not adjusted for sex. Linear, cubic, and quadratic regression plots were presented for each cumulative ST, LPA, and MVPA progression (ages 11–24 years) with each of the LVMI^2.7^, RWT, LVDF, and LVFP progression at ages 17–24 years to visualize the adequacy of the linearity assumption (*Figure 2* and Supplementary material online, *Figures S1*–*S3*).

To assess the dose–response relationships of MVPA with each of the progression in LVMI^2.7^, RWT, LVDF, and LVFP measured from ages 17 through 24 years using GLMMs for repeated measures, the MVPA established cutpoints^9^ of ≥60 min/day, 40– < 60 min/day, and <40 min/day as the reference category were computed at ages 11, 15, and 24 years, respectively. Each category at baseline (age 11 years) was combined with their corresponding categories at ages 15- and 24-year follow-up in a restructured format. The MVPA categorization resulted in persistent ≥60 min/day, persistent 40– <60 min/day, and persistent <40 min/day from ages 11 through 24 years.

Analyses were repeated in a subset of participants with overweight and obesity and elevated and hypertensive systolic blood pressure. Moreover, among 766 participants with at least two timepoint complete cardiac measures of ST, LPA, and MVPA at 17 years the longitudinal analyses were repeated and are presented in the Supplementary material online, Appendix. Sequential Sidak correction was applied to correct for multiple comparisons. Covariates were selected based on previous studies.^29,36,37,44,47^ Collinearity diagnoses were performed and accepted results with a variance inflation factor <5. Differences and associations with a two-sided *P*-value <0.05 were considered statistically significant, and conclusions were made based on effect estimates and their CIs. The effect estimate for continuous predictor variable analyses describes a 1-min/day change in ST, LPA, or MVPA over 13 years in relation to the computed point estimate unit change in cardiac measure over 7 years. The effect estimates from the categorical predictor variable describe a persistent MVPA higher category in contrast to the reference or lowest category over 13 years in relation to the computed point estimate unit change in cardiac measure over 7 years. Analyses involving 20% of a sample of 10 000 ALSPAC children at 0.8 statistical power, 0.05 alpha, and two-sided *P*-value would show a minimum detectable difference of 0.062 standard deviations if they had relevant exposure for a normally distributed quantitative variable.^48^ All statistical analyses and multiple imputations were performed using SPSS statistics software, version 27.0 (IBM Corp, Armonk, NY, USA).

## Results

### Cohort study characteristics

Altogether, 1682 (62.7% female) participants who had at least one-time measures of ST, LPA, and MVPA during the ages 11–24-year observation period and complete LVMI^2.7^, RWT, LVDF, and LVFP measurements at the age of 24-year clinic visit were studied (*Figure 1*). Males had ∼+4 g/m^2.7^ of LVM than females at both baseline and follow-up, while RWT, LVDF, and LVFP were similar in both sexes (*Table 1*). RWT decreased, while LVDF and LVFP increased over 7 years. Females had higher ST from 11 to 24 years, whereas males accumulated more MVPA minutes (*Table 1*). Other characteristics are described in *Table 1*.

**Table 1:** Descriptive characteristics of cohort participants

	17 years			24 years		
Variables	Male (*n* = 577)	Female (*n* = 970)	*P*-value	Male (*n* = 628)	Female (*n* = 1054)	*P*-value
	Mean (SD)	Mean (SD)		Mean (SD)	Mean (SD)	
***Anthropometry***						
Age at clinic visit (years)	17.73 (0.36)	17.74 (0.39)	0.574	24.38 (0.58)	24.34 (0.62)	0.211
Age at 11-year clinic visit	11.75 (0.24)	11.75 (0.24)	0.381	NA		
Height (m)	1.79 (0.07)	1.65 (0.06)	<0.0001	1.80 (0.07)	1.66 (0.06)	<0.0001
*Weight (kg)	68.95 (13.40)	59.80 (12.70)	<0.0001	76.6 (16.47)	63.80 (14.90)	<0.0001
Ethnicity—White (*n*, %)	573 (96.8)	932 (95.8)	0.345	NA		
***Body composition***						
*Total fat mass (kg)	10.08 (8.84)	18.81 (9.65)	<0.0001	17.27 (9.67)	21.27 (10.66)	<0.001
*Lean mass (kg)	54.70 (8.18)	37.74 (5.09)	<0.0001	55.70 (10.33)	40.45 (6.51)	<0.0001
*Body mass index (kg/m^2^)	21.24 (3.76)	21.76 (4.02)	0.010	23.40 (4.29)	23.10 (4.95)	0.167
Overweight/obese body mass index ≥25 kg/m^2^ (n, %)	79 (14.1)	174 (18.3)	0.037	208 (33.1)	326 (31.0)	0.357
***Fasting plasma metabolic indices***						
High-density lipoprotein (mmol/L)	1.20 (0.26)	1.38 (0.31)	<0.0001	1.42 (0.36)	1.69 (0.43)	<0.0001
Low-density lipoprotein (mmol/L)	2.00 (0.58)	2.24 (0.65)	<0.0001	2.37 (0.74)	2.37 (0.73)	0.968
*Triglyceride (mmol/L)	0.73 (0.31)	0.74 (0.35)	0.351	0.86 (0.51)	0.79 (0.42)	<0.0001
Glucose (mmol/L)	5.15 (0.44)	4.91 (0.38)	<0.0001	5.55 (0.96)	5.22 (0.49)	<0.0001
*Insulin (mU/L)	5.66 (3.48)	7.29 (4.51)	<0.0001	6.90 (4.46)	7.68 (5.29)	0.001
*High-sensitivity C-reactive protein (mg/L)	0.40 (0.53)	0.59 (1.24)	<0.0001	0.59 (1.12)	0.95 (1.93)	<0.0001
***Vascular measures***						
Heart rate (beat/min)	63 (9)	67 (10)	<0.0001	64 (10)	68 (10)	<0.0001
Systolic blood pressure (mmHg)	119 (9)	110 (8)	<0.0001	122 (10)	111 (10)	<0.0001
Elevated/hypertensive blood pressure ≥130 mmHg (n, %)	62 (11.3)	<7 (0.5)	<0.0001	123 (19.6)	43 (4.1)	<0.0001
Diastolic blood pressure (mmHg)	63 (7)	65 (6)	<0.0001	67 (7)	66 (8)	0.101
***Cardiac measures***						
Left ventricular mass indexed for height (g/m^2.7^)	36.94 (8.01)	34.54 (6.97)	<0.0001	40.87 (8.91)	36.61 (7.86)	<0.0001
Relative wall thickness (cm)	0.38 (0.06)	0.38 (0.06)	0.267	0.37 (0.05)	0.36 (0.06)	0.003
Left ventricular diastolic function (E/A)	1.95 (0.38)	1.92 (0.38)	0.311	1.96 (0.52)	2.01 (0.61)	0.077
Left ventricular filling pressure (E/eʹ)	4.79 (1.16)	4.87 (0.90)	0.234	4.86 (0.98)	5.16 (1.04)	<0.0001
***Lifestyle factors***						
Smoked cigarette in the last 30 days (*n*, %)	105 (21.2)	203 (24.3)	0.192	164 (26.5)	266 (25.4)	0.608
Family history of H–D–C–V (*n*, %)	152 (27.4)	293 (31.6)	0.090	NA		
Sedentary time at 11 years (min/day)	359 (70)	368 (73)	0.014	NA		
Sedentary time at 15–24 years (min/day)	466 (86)	486 (77)	<0.0001	528 (81)	523 (86)	0.450
Cumulative sedentary time from 11–24 years (min/day)	424 (104)	436 (103)	<0.0001	NA		
Light-intensity physical activity at 11 years (min/day)	366 (60)	364 (60)	0.494	NA		
Light-intensity physical activity at 15–24 years (min/day)	287 (68)	270 (61)	<0.0001	146 (58)	148 (53)	0.528
Cumulative light-intensity physical activity from 11 to 24 years (mins/day)	300 (105)	290 (104)	<0.0001	NA		
Moderate-to-vigorous-intensity physical activity at 11 years (min/day)	66 (32)	46 (20)	<0.0001	NA		
Moderate-to-vigorous-intensity physical activity at 11 years 40–<60 min/day (*n*, %)	153 (27.1)	315 (33.1)	<0.001	NA		
Moderate-to-vigorous-intensity physical activity at 11 years ≥60 min/day (*n*, %)	307 (54.3)	213 (22.4)	<0.001	NA		
Moderate-to-vigorous-intensity physical activity at 15–24 years (min/day)	55 (32)	39 (21)	<0.0001	55 (34)	47 (29)	0.002
Moderate-to-vigorous-intensity physical activity at 15–24 years 40–<60 min (*n*, %)	86 (28.2)	145 (27.5)	<0.001	56 (24.6)	113 (26.0)	0.007
Moderate-to-vigorous-intensity physical activity at 15–24 years ≥60 min/day (*n*, %)	116 (38.0)	85 (16.1)	<0.001	84 (36.8)	116 (26.7)	0.007
Cumulative moderate-to-vigorous-intensity physical activity from 11–24 years (min/day)	60 (33)	45 (23)	<0.0001	NA		
Maternal social economic status (*n*, %)			0.588	NA		
*Professional*	36 (12.2)	27 (5.5)				
*Managerial and technical*	114 (38.8)	208 (42.1)				
*Skilled non-manual*	90 (30.6)	178 (36.0)				
*Skilled manual*	<7 (1.4)	9 (1.8)				
*Partly skilled*	41 (13.9)	62 (12.6)				
*Unskilled*	9 (3.1)	10 (2.0)				

The values are means (standard deviations) and *median (inter-quartile range) except for lifestyle factors and ethnicity. Differences between sexes were tested using Student’s *t*-test for normally distributed continuous variables, Mann–Whitney U test for skewed continuous variables, χ^2^ test for dichotomous variables, and analysis of covariance for the multicategory variable. A two-sided *P* <0.05 is considered statistically significant. NA, not available/applicable; *P*-value for sex differences. H–D–C–V, hypertension, diabetes, high cholesterol, and vascular disease.

### Longitudinal associations of ST, LPA, and MVPA during ages 11–24 years with changes in cardiac structure and function during ages 17–24 years

The cumulative increase in ST from ages 11 to 24 years was associated with progressively increased LVMI^2.7^ from ages 17 to 24 years after full adjustments in the whole cohort and among males and females (*Tables 2* and *3*). The cumulative increase in ST was associated with increased change in RWT and LVFP among males and increased LVFP among females (*Table 3*). The statistically significant relationship between the cumulative increase in ST and increased change in LVDF in males and RWT and LVDF in females was attenuated after further adjustments for cumulative LPA and MVPA (*Table 3*). Cumulative LPA was associated with a decreased change in LVMI^2.7^ but increased RWT and LVDF in the whole cohort and males (*Tables 2* and *3*). Cumulative LPA was associated with increased LVMI^2.7^, RWT, LVDF, and LVFP in females (*Table 3*).

**Table 2:** Longitudinal associations of cumulative sedentary time and physical activity from ages 11 through 24 years with the progressive change in left ventricular mass and diastolic function from ages 17 through 24 years

*n* = 1682	LVMI^2.7^(g/m^2.7^)		RWT (cm)		LVDF (E/A)		LVFP (E/eʹ)	
	*β (95% CI)*	*P-value*	*β (95% CI)*	*P-value*	*β (95% CI)*	*P-value*	*β (95% CI)*	*P-value*
***Continuous cumulative predictor variables from ages 11 to 24 years***								
**Sedentary time**								
*Model 1*	0.006 (0.005–0.006)	**<0**.**0001**	−0.000 (−0.000 to −0.000)	**<0**.**0001**	0.000 (0.000–0.000)	**<0**.**0001**	0.000 (0.000–0.00002)	0.114
*Model 2*	0.002 (0.001–0.003)	**<0**.**001**	0.000 (−0.000–0.000)	0.156	0.000 (−0.000–0.000)	0.278	0.000 (0.000–0.000)	0.642
**Light-intensity physical activity**								
*Model 1*	−0.006 (−0.007 to −0.005)	**<0**.**0001**	0.00001 (0.000–0.000)	**<0**.**0001**	0.000 (0.000–0.000)	**<0**.**0001**	0.000 (0.0001–0.000)	**0**.**029**
*Model 2*	−0.005 (−0.006 to −0.003)	**<0**.**0001**	0.000 (0.000–0.000)	**<0**.**0001**	0.000 (0.000–0.001)	**<0**.**0001**	−0.000 (0.000–0.000)	0.645
**Moderate-to-vigorous-intensity physical activity**								
*Model 1*	0.001 (−0.002–0.003)	0.492	0.000 (−0.000–0.000)	0.428	0.000 (0.000–0.0001)	0.241	0.000 (0.000–0.001)	0.485
*Model 2*	0.003 (0.001–0.006)	**0**.**015**	−0.000 (−0.000–0.000)	0.088	0.000 (0.001 to −0.000)	0.161	0.000 (0.000–0.000)	0.610
***Categorical cumulative predictor variable from ages 11 to 24 years***								
**Moderate-to-vigorous-intensity physical activity (*<40 min/day as reference)***								
*40–<60 mins/day*	−0.232 (−0.460 to −0.003)	**0**.**047**	−0.001 (−0.004–0.001)	0.191	−0.021 (−0.038 to −0.005)	**0**.**013**	−0.007 (−0.041–0.027)	0.674
*≥60 mins/day*	0.054 (−0.135–0.244)	0.574	0.0001 (−0.002 to −0.002)	0.914	−0.019 (−0.033 to −0.005)	**0**.**007**	0.029 (0.000–0.057)	0.050

Model 1 was adjusted for sex, age at baseline, and other time-varying covariates measured at both baseline and follow-up such as low-density lipoprotein cholesterol, insulin, triglyceride, high-sensitivity C-reactive protein, high-density lipoprotein cholesterol, heart rate, systolic blood pressure, glucose, fat mass, lean mass, smoking status, family history of hypertension/diabetes/high cholesterol/vascular disease, and socioeconomic status. Model 2 was additional adjustment for sedentary time, light-intensity physical activity, or moderate-to-vigorous-intensity physical activity depending on the predictor. For categorical predictor variable analyses, all the above-listed covariates were adjusted for in one model. Skewed covariates were logarithmically transformed. Regression coefficients *(**β)*** were computed from generalized linear mixed-effect model for repeated measures. A two-sided *P* <0.05 is considered statistically significant and bolded. Multiple testing was corrected with Sidak correction. Multiple imputations were used to account for missing variables. For continuous variable predictors (ST, LPA, and MVPA), a 1-min change is associated with the point estimate unit change in the outcome. For categorical variable predictor (MVPA), time spent in a category in relation to the reference is associated with the point estimate unit change in the outcome.

CI, confidence interval; LVDF, left ventricular diastolic function; LVFP, left ventricular filling pressure; LVMI^2.7^, left ventricular mass indexed for height^2.7^; RWT, relative wall thickness.

**Table 3:** Longitudinal associations of cumulative sedentary time and physical activity from ages 11 through 24 years with the progressive change in left ventricular mass and diastolic function from ages 17 through 24 years according to sex

	LVMI^2.7^ (g/m^2.7^)		RWT (cm)		LVDF (E/A)		LVFP (E/e´)	
	*β (95% CI)*	*P-value*	*β (95% CI)*	*P-value*	*β (95% CI)*	*P-value*	*β (95% CI)*	*P-value*
***Male (n = 628)***								
**Sedentary time**								
*Model 1*	0.011 (0.010–0.013)	**<0**.**0001**	−0.000 (−0.000 to −0.000)	**<0**.**001**	0.000 (0.000–0.000)	**<0**.**001**	0.000 (0.000–0.000)	0.327
*Model 2*	0.002 (0.000–0.004)	**0**.**014**	0.000 (0.000–0.000)	**<0**.**001**	0.000 (−0.000–0.000)	0.307	0.001 (0.000–0.001)	**<0**.**001**
**Light-intensity physical activity**								
*Model 1*	−0.014 (−0.015 to −0.012)	**<0**.**0001**	0.000 (0.000–0.000)	**<0**.**001**	0.000 (0.000–0.001)	**<0**.**0001**	0.000 (0.000 to −0.000)	0.252
*Model 2*	−0.012 (−0.014 to −0.010)	**<0**.**0001**	0.000 (0.000–0.000)	**<0**.**001**	0.000 (0.000–0.001)	**<0**.**001**	0.000 (0.000–0.001)	**0**.**003**
**Moderate to vigorous physical activity**								
*Model 1*	−0.011 (−0.015 to −0.007)	**<0**.**001**	0.000 (0.000–0.000)	**0**.**002**	0.001 (0.000–0.001)	**<0**.**001**	0.001 (0.000–0.001)	**0**.**030**
*Model 2*	0.001 (−0.003–0.005)	0.670	0.000 (−0.000–0.000)	0.125	0.000 (0.000–0.001)	**0**.**020**	0.001 (0.000–0.001)	**0**.**008**
**Categorical cumulative predictor variable from ages 11 to 24 years**								
***Moderate-to-vigorous-intensity physical activity (<40 min/day as reference)***								
*40–<60 min/day*	−0.415 (−0.755 to −0.755)	**0**.**017**	−0.003 (−0.006–0.000)	**0**.**042**	−0.039 (−0.061 to −0.017)	**<0**.**001**	0.015 (−0.035–0.065)	0.554
*≥60 min/day*	−0.325 (−0.653–0.003)	0.052	−0.002 (−0.005–0.001)	0.212	0.005 (−0.015–0.025)	0.644	0.073 (0.022–0.123)	**0**.**005**
***Female (n = 1054)***								
**Sedentary time**								
*Model 1*	0.001 (0.000–0.002)	**0**.**047**	−0.0001 (−0.0001 to −0.0001)	**<0**.**0001**	0.000 (0.000 to −0.000)	**0**.**011**	0.000 (−0.000–0.000)	0.063
*Model 2*	0.002 (0.001–0.003)	**<0**.**001**	0.0001 (−0.0001–0.0001)	0.470	−0.000 (0.000–0.000)	0.591	0.001 (0.000 to −0.001)	**0**.**013**
**Light-intensity physical activity**								
*Model 1*	0.000 (0.000–0.0001)	<0.0001	0.000 (0.000–0.0001)	**<0**.**0001**	0.000 (0.000–0.000)	**0**.**003**	0.000 (−0.001–0.000)	**<0**.**001**
*Model 2*	0.002 (0.001–0.003)	**0**.**001**	0.000 (0.000–0.0001)	**<0**.**0001**	0.000 (0.001–0.0001)	**0**.**031**	−0.001 (−0.001–0.000)	**<0**.**001**
**Moderate-to-vigorous-intensity physical activity**								
*Model 1*	−0.007 (−0.010 to −0.003)	**<0**.**001**	−0.000 (−0.000 to −0.000)	**0**.**007**	0.000 (0.001–0.001)	0.205	0.001 (0.000–0.001)	**0**.**005**
*Model 2*	−0.006 (−0.009 to −0.002)	**0**.**001**	−0.000 (0.000 to −0.000)	**<0**.**001**	0.000 (0.001–0.001)	0.431	0.001 (0.000–0.001)	**0**.**005**
**Categorical cumulative predictor variable from ages 11 to 24 years**								
***Moderate-to-vigorous-intensity physical activity (<40 min/day as reference)***								
*40–<60 min/day*	−0.363 (−0.612 to −0.115)	**0**.**004**	0.000 (−0.002–0.003)	0.928	0.001 (−0.018–0.021)	0.896.	−0.017 (−0.056–0.023)	0.411
*≥60 min/day*	−0.382 (−0.609 to −0.154)	**0**.**001**	−0.004 (−0.006 to −0.002)	**<0**.**001**	0.008 (−0.010–0.026)	0.356	0.043 (0.010–0.076)	**0**.**011**

Model 1 was adjusted for age at baseline and other time-varying covariates measured at both baseline and follow-up such as low-density lipoprotein cholesterol, insulin, triglyceride, high-sensitivity C-reactive protein, high-density lipoprotein cholesterol, heart rate, systolic blood pressure, glucose, fat mass, lean mass, smoking status, family history of hypertension/diabetes/high cholesterol/vascular disease, and socioeconomic status. Model 2 was additional adjustment for sedentary time, light-intensity physical activity, or moderate-to-vigorous-intensity physical activity depending on the predictor. Skewed covariates were logarithmically transformed. Regression coefficients *(**β**)* were computed from generalized linear mixed-effect model for repeated measures. A two-sided *P* <0.05 is considered statistically significant and bolded. Multiple testing was corrected with Sidak correction. Multiple imputations were used to account for missing variables. For continuous variable predictors (ST, LPA, and MVPA), a 1-min change is associated with the point estimate unit change in the outcome.

CI, confidence interval; LVDF, left ventricular diastolic function; LVFP, left ventricular filling pressure; LVMI^2.7^, left ventricular mass indexed for height^2.7^; RWT, relative wall thickness.

Cumulative MVPA was associated with increased changes in LVMI^2.7^ in the whole cohort but increased LVDF and LVFP in males (*Tables 2* and *3*). Among females, cumulative MVPA was associated with decreased LVMI^2.7^ and RWT and increased LVFP. In the whole cohort, persistent MVPA of ≥60 min/day from ages 11 to 24 years was associated with decreased LVDF (*Table 2*). Persistent MVPA of 40–≤60 min/day from ages 11 to 24 years was associated with decreased LVMI^2.7^ and LVDF (*Table 2*). Among males, persistent MVPA of ≥60 min/day from ages 11 to 24 years was associated with decreased LVFP; however, persistent MVPA of 40– ≤60 min/day from ages 11 to 24 years was associated with decreased LVMI^2.7^, RWT, and LVDF (*Table 3*). Among females, persistent MVPA of ≥60 min/day from ages 11 to 24 years was associated with decreased LVMI^2.7^ and RWT and increased LVFP; however, persistent MVPA of 40–≤60 min/day from ages 11 to 24 years was associated with decreased LVMI^2.7^only (*Table 3*). The unadjusted longitudinal associations of ST, LPA, and MVPA with LVMI^2.7^, RWT, LVDF, and LVFP in the total cohort are presented in *Figure 2* and Supplementary material online, *Figures S1*–*S3*.

In a subset of 766 participants who had at least two timepoints complete measures of ST, LPA, and MVPA during ages 11–24-year clinic visits with complete cardiac measures at 17 years, the results were largely consistent in that cumulative ST and MVPA were independently associated with progressively increased LVMI^2.7^, while LPA was associated with decreased LVMI^2.7^ in the total cohort and males (see Supplementary material online, *Tables S1* and *S2*). Among females, cumulative ST and LPA were associated with increased LVMI^2.7^, while MVPA was associated with decreased LVMI^2.7^.

### Longitudinal associations of ST, LPA, and MVPA with cardiac structure and function based on overweight/obesity and elevated/hypertensive blood pressure status at age 24 years

Among participants who were overweight and obese at age 24 years, cumulative ST was associated with increased LVMI^2.7^, RWT, and LVFP after full adjustment (*Table 4*). Cumulative LPA was associated with decreased LVMI^2.7^, RWT, and LVFP but increased LVDF. Cumulative MVPA had no significant associations with all cardiac measures (*Table 4*).

**Table 4:** Longitudinal associations of cumulative sedentary time and physical activity from ages 11 through 24 years with the progressive change in left ventricular mass and diastolic function from ages 17 through 24 years according to overweight/obesity and elevated blood pressure status at age 24 years

	LVMI^2.7^(g/m^2.7^)		RWT (cm)		LVDF (E/A)		LVFP (E/eʹ)	
	*β (95% CI)*	*P-value*	*β (95% CI)*	*P-value*	*β (95% CI)*	*P-value*	*β (95% CI)*	*P-value*
***Overweight/obese ≥25 kg/m^2^ (n = 535)***								
**Sedentary time**								
*Model 1*	0.011 (0.009–0.013)	**<0**.**0001**	0.000 (0.000–0.000)	**<0**.**001**	0.000 (0.000–0.001)	**<0**.**001**	0.001 (0.001–0.001)	**<0**.**001**
*Model 2*	0.006 (0.003–0.009)	**<0**.**001**	0.000 (0.000–0.000)	**0**.**002**	0.000 (0.000–0.000)	0.566	0.000 (0.000–0.001)	**0**.**040**
**Light-intensity physical activity**								
*Model 1*	−0.011 (−0.014 to −0.009)	**<0**.**0001**	−0.000 (−0.000 to −0.000)	**<0**.**001**	−0.001 (−0.001–0.000)	**<0**.**001**	−0.001 (−0.002 to −0.001)	**<0**.**001**
*Model 2*	−0.007 (−0.010 to −0.004)	**<0**.**001**	−0.000 (−0.000 to −0.000)	**0**.**011**	0.000 (−0.000–0.001)	**<0**.**001**	−0.001 (−0.001 to −0.001)	**<0**.**001**
**Moderate to vigorous physical activity**								
*Model 1*	−0.005 (−0.013–0.004)	0.298	−0.000 (0.000–0.000)	0.148	−0.001 (−0.001–0.000)	**0**.**006**	−0.001 (−0.002–0.000)	0.062
*Model 2*	0.004 (−0.005–0.013)	0.347	−0.000 (−0.000–0.000)	0.947	0.000 (−0.000–0.000)	0.200	0.000 (−0.001–0.001)	0.825
***Elevated and hypertensive systolic blood pressure ≥130 mmHg (n = 170)***								
**Sedentary time**								
*Model 1*	0.012 (0.008–0.016)	**<0**.**001**	0.000 (0.0001–0.000)	**<0**.**001**	0.000 (0.000–0.001)	**0**.**002**	0.001 (0.000–0.001)	**<0**.**001**
*Model 2*	0.006 (0.001–0.011)	**0**.**011**	0.0001 (0.0001–0.000)	**<0**.**001**	0.000 (0.000–0.001)	**<0**.**001**	0.002 (0.001–0.002)	**<0**.**001**
**Light-intensity physical activity**								
*Model 1*	−0.014 (−0.018 to −0.009)	**<0**.**001**	0.000 (0.000 to −0.0001)	**<0**.**001**	0.000 (0.000–0.000)	0.282	0.000 (0.000–0.001)	0.675
*Model 2*	−0.010 (−0.015 to −0.005)	**<0**.**001**	−0.000 (−0.000–0.0001)	0.076	0.000 (0.001–0.0001)	0.276	0.001 (0.001–0.002)	**<0**.**001**
**Moderate-to-vigorous-intensity physical activity**								
*Model 1*	−0.003 (−0.016–0.011)	0.674	−0.000 (0.000–0.000)	0.099	−0.001 (−0.001 to −0.0001)	**0**.**037**	0.001 (−0.001–0.003)	0.277
*Model 2*	0.005 (−0.008–0.018)	0.466	−0.000 (0.000–0.000)	0.638	0.000 (−0.001–0.000)	0.112	0.002 (0.000–0.004)	0.101

Model 1 was adjusted for age at baseline and other time-varying covariates measured at both baseline and follow-up such as low-density lipoprotein cholesterol, insulin, triglyceride, high-sensitivity C-reactive protein, high-density lipoprotein cholesterol, heart rate, glucose, smoking status, family history of hypertension/diabetes/high cholesterol/vascular disease, socioeconomic status in addition to systolic blood pressure and fat mass, lean mass depending on overweight or hypertensive category. Model 2 was additional adjustment for sedentary time, light-intensity physical activity, or moderate-to-vigorous--intensity physical activity depending on the predictor. Skewed covariates were logarithmically transformed. Regression coefficients *(**β)*** were computed from generalized linear mixed-effect model for repeated measures. A two-sided *P* <0.05 is considered statistically significant and bolded. Multiple testing was corrected with Sidak correction. Multiple imputations were used to account for missing variables. For continuous variable predictors (ST, LPA, and MVPA), a 1-min change is associated with the point estimate unit change in the outcome.

CI, confidence interval; LVDF, left ventricular diastolic function; LVFP, left ventricular filling pressure; LVMI^2.7^, left ventricular mass indexed for height^2.7^; RWT, relative wall thickness.

Among participants with elevated and hypertensive blood pressure at age 24 years, cumulative ST was associated with increased LVMI^2.7^, RWT, LVDF, and LVFP (*Table 4*). Cumulative LPA was associated with decreased LVMI^2.7^ and increased LVFP. Cumulative MVPA had no significant associations with all cardiac measures (*Table 4*).

## Discussion

In the largest and longest follow-up study to date, accelerometer-measured cumulative ST, LPA, and MVPA from childhood through young adulthood were independently but differently related to the changes in cardiac structure and function during growth. Cumulatively increased ST from childhood through young adulthood was associated with increased cardiac mass, whereas cumulative LPA from childhood was associated with decreased cardiac mass. Paradoxically, cumulative MVPA was associated with increased cardiac mass and decreased cardiac function. Similarly, persistent MVPA ≥60 min/day was associated with decreased cardiac function in young adulthood, but persistent MVPA of 40–<60 min/day was associated with decreased cardiac mass.

### Cumulative ST with changes in cardiac structure and function

Disparities exist in the role of self-reported sedentary behaviour on adverse cardiometabolic health in youth in comparison with device-measured ST.^9,10,22^ While the former associates with worsening cardiometabolic health, the latter (device-measured ST) showed no consistent relationships.^9^ There is a paucity of evidence on device-based measured ST in relation to cardiac function and structure in the paediatric population.^9^ This present longitudinal study revealed that average ST increased by almost 3 h from childhood (ST = 6 h) through young adulthood (ST = 9 h) over 13 years. Likewise, LVMI increased on average by 3 g/m^2.7^ during growth from adolescence to young adulthood. Each 1-min increase in ST from childhood through young adulthood was associated with a 0.002 (0.001–0.003) g/m^2.7^ LVMI increase over 7 years. This suggests that cumulative ST of 431 min/day on average may maximally account for 40% of the increase in cardiac mass, i.e. +1.29 g/m^2.7^ of 3 g/m^2.7^ from adolescence through young adulthood. This is consistent with findings from a recent cross-sectional report where higher ST was associated with a 30% higher rise in LVMI in 17-year-old adolescents.^23^ There was strong consistency in the longitudinal relationships between cumulative ST and increased cardiac mass irrespective of sex, obesity, and hypertensive status. The ST-related increase in LVMI in both cross-sectional and longitudinal study designs may be clinically significant since among adults, an ∼5 g/m^2^ higher LVMI may correspond to a 7–20% increase in CVD morbidity and mortality.^49^ Progressively increasing mitral E/A ratio (LVDF) and E/eʹ ratio (LVFP) predict heart failure with preserved and reduced ejection fraction, acute myocardial infarction, and cardiovascular and all-cause mortality.^36,37,47,50^ Cumulative ST from childhood was associated with the progressive increase in LVFP in males, females, and among participants who were overweight/obese or with elevated and hypertensive blood pressure. It was recently shown that increased ST from childhood may worsen blood pressure and accelerate premature vascular damage.^14,51^ These findings reveal that the deleterious effect of childhood ST on cardiovascular structure and function may be evident from adolescence.

### Cumulative LPA with changes in cardiac structure and function

In the latest health guideline, there was no recommendation on LPA, due to the scarcity of evidence on device-based measured LPA in youth.^9^ Moreover, the relationships between LPA and cardiac indices in the paediatric population are unclear.^10^ It has been established that most youths do not meet the recommended average of 60 min of MVPA per day but may accumulate a substantial amount of LPA minutes per day.^16^ In the present longitudinal study, cumulative LPA was independently associated with decreased cardiac mass and LVFP. Importantly, time spent in LPA in childhood (∼6 h) decreased by nearly 60% by young adulthood. Nonetheless, each 1 min of cumulative LPA was associated with a −0.005 g/m^2.7^ decrease in cardiac mass over a 7-year period, independent of ST, MVPA, and cardiometabolic and lifestyle factors. A 3 g/m^2.7^ increase in LVMI during the 7-year follow-up period was observed, and the cumulative LPA exposure was 292 min/day on average during growth from childhood through young adulthood. Thus, cumulative LPA may decrease LVMI by −1.46 g/m^2.7^, amounting to a circa 49% reduction in the increase in LVMI over 7 years. In a cross-sectional report, LPA had no statistically significant association with cardiac mass but with better cardiac function and LPA was inversely associated with arterial stiffness among adolescents.^15,23^ The longitudinal relationships between cumulative LPA and decreased cardiac mass in males among participants who are overweight/obese or with elevated and hypertensive blood pressure were consistently strong. These findings suggest that accumulating more LPA times may significantly attenuate the deleterious effects of ST on cardiac mass. Hence, LPA may be prescribed to enhance better cardiac health in the paediatric population, even in populations at risk of obesity and hypertension.^24,29,52,53^ On the contrary, among females, cumulative LPA was associated with increased LVMI, RWT, and LVDF but decreased LVFP, suggesting that LPA-related cardiac benefits may differ slightly by sex probably because of inherent physiology, but further mechanistic studies are needed to clarify these differences.

### Cumulative MVPA with changes in cardiac structure and function

Cumulative MVPA from childhood was paradoxically associated with progressively increased LVM from ages 17 to 24 years in the total cohort only. Moreover, a persistent ≥60 min/day of MVPA was not associated with the progressive increase in cardiac mass either in the total cohort, in males, and among participants who were overweight/obese or had elevated or hypertensive blood pressure. Increased LVM in adults predicts higher risks of cardiovascular morbidity and mortality.^1,2,47^ Importantly, accumulating 40–<60 min/day of MVPA from childhood in relation to <40 min/day of MVPA was associated with a 0.23 g/m^2.7^ decreased LVMI in the total cohort and irrespective of sex across the 7-year repeated cardiac assessment. The combined effect of 40–<60 min/day of MVPA and LPA may be clinically significant since a 25.3 unit decrease in LVMI was associated with a 22% less likelihood of cardiovascular morbidity and mortality.^2^

Cardiac wall stretch from pressure overload results in concentric remodelling or hypertrophy with no change in the size of the cardiac internal cavity.^54^ Cardiac volume overload leads to eccentric remodelling or hypertrophy with changes in the cardiac internal cavity.^54^ Normal end-diastolic volume and an increased RWT suggest concentric hypertrophy if LVM is increased and concentric remodelling if LVM is normal.^54^ High end-diastolic volume and decreased RWT suggest eccentric hypertrophy if LVM is increased and eccentric remodelling if LVM is normal.^54^ In youth athletes, LV dilatation and mild concentric LV hypertrophy have been reported in cardiac adaptation to exercise training.^55^ In normal living 15–17-year-old adolescents, higher MVPA was associated with 10% higher LVMI.^23^ This contrasts adult athletes’ cardiac adaptation to exercise where less chamber dilatation and eccentric hypertrophy tend to occur.^55^ However, both youth and adult athletes exhibit similar increases in LV relaxation and improved LVDF.^55^ The present longitudinal findings in a resting state may indicate increasing chamber dilatation, LV relaxation, and increasing cardiac mass, reflecting a gradual transition from concentric remodelling to concentric hypertrophy.^54,55^ There was a two-fold increase in LVMI in males (∼4 g/m^2.7^) compared with females (∼2 g/m^2.7^) from ages 17 through 24 years; in addition, males accumulated 15–20 min more MVPA time than females during the 13-year observation period. Thus, it appears that the relatively lesser amount of time females spent in MVPA was paradoxically associated with decreased cardiac mass. These findings suggest a sex-based tailored approach to MVPA interventions for cardiac health benefits in youth.

Among participants with either overweight/obesity or elevated blood pressure, cumulative MVPA had no statistically significant relationship with cardiac structural changes. It was recently shown that increased fat mass dampens the effect of MVPA on metabolic indices such as lipid and inflammation.^11–13^ It has also been reported that MVPA significantly contributes to an increase in lean mass which is a driver of cardiac mass increase.^11,38^ Recent studies have reported that MVPA’s positive association with increased vascular stiffness is due to the mediating effect of lean mass.^14^ Taken together, the effect of body composition on MVPA and cardiac outcomes increases the likelihood of MVPA’s null effect in its relationship with cardiac structure among participants who are overweight or obese since these participants have significantly high fat mass and high lean mass.^11,41^ In addition, MVPA may paradoxically increase blood pressure by increasing lean mass, which might explain the null findings between MVPA and cardiac structural changes in participants with elevated blood pressure.^51^ Nonetheless, in the main analyses, blood pressure, fat mass, and lean mass were adjusted for as well as other covariates, yet cumulative MVPA was associated with increased LVMI. Increased ST has been associated with increased fat mass, blood pressure, inflammation, and elevated lipid levels which are independent causal risk factors for increased LVMI and premature cardiac damage in youth.^6,8,11,12,51^ ST also paradoxically increases lean mass.^11^ However, MVPA decreases fat mass and slightly lowers lipid levels and inflammation but might slightly raise blood pressure and increase lean mass.^11,13,51^ Cumulative ST from childhood maximally contributed +40% (+1.29 out of the total 7-year increase 3 g/m^2.7^) in LVMI from adolescence to young adulthood. This is at least eight-fold more than MVPA-induced LVMI increase. Each minute of cumulative MVPA from childhood was associated with progressively increased LVMI (+0.003 g/m^2.7^), amounting to +0.15 out of 3 g/m^2.7^ which is an approximate +5% increase in LVMI. Thus, MVPA-induced LVMI increase seems lean mass–driven physiological remodelling. On the contrary, ST-induced LVMI increase appears fat mass, lipid, inflammation, and blood pressure–driven pathologic remodelling, which is of clinical and public health importance.^25^

### Strength and limitations

From a well-phenotyped large birth cohort (ALSPAC) with extensive repeated assessments from childhood through young adulthood, a comprehensive array of repeatedly measured covariates such as device-measured body composition such as fat mass, lean mass, and other lifestyle factors smoking, socioeconomic status, and family history of cardiovascular and metabolic diseases, it was possible to examine the independent relationships of movement behaviour with cardiac indices. A few limitations are that accelerometer was worn for 7 days, which may not be sufficient to reveal the habitual lifestyle of the participants. The possibility of a Hawthorne effect cannot be excluded where participants modify their behaviour based on their awareness of being observed. Nonetheless, the device-measured PA and ST is superior to self-reported lifestyle behaviour which is fraught with recall and report bias.^22^ Some participants with cardiac structural and functional measures at baseline did not meet the inclusion criteria because they lacked PA measures. Nonetheless, these participants had similar baseline metabolic and cardiac characteristics as those included in the present study (data not shown). Also, participants were mostly Caucasian; thus, findings may not be generalizable to other ethnicities. Residual biases such as not accounting for sleep time may not be excluded since the measures were not available.

## Conclusions

Increasing LPA and decreasing ST may independently attenuate and reverse progressively worsening changes in cardiac structure and function from adolescence through young adulthood. Cumulative MVPA and spending ≥60 min of MVPA/day were paradoxically associated with increased cardiac mass and decreased cardiac function, especially in males. LPA may be prescribed and recommended in the paediatric population, especially for those with disease conditions such as hypertension and obesity and who are unwilling to participate in vigorous exercises since LPA could potentially reverse worsening cardiac indices associated with ST. ST-induced LVMI increase was eight times higher than MVPA-included LVMI increase during the growth from adolescence to young adulthood, suggesting ST-induced cardiac pathologies. These findings may be considered in updating future health guidelines.

## Supplementary Material

zwae129_Supplementary_Data

## Data Availability

The informed consent obtained from ALSPAC participants does not allow the data to be made freely available through any third-party maintained public repository. However, data used for this submission can be made available on request to the ALSPAC Executive. The ALSPAC data management plan describes in detail the policy regarding data sharing, which is through a system of managed open access. Full instructions for applying for data access can be found here: http://www.bristol.ac.uk/alspac/researchers/access/. The ALSPAC study web site contains details of all the data that are available (http://www.bristol.ac.uk/alspac/researchers/our-data/).

## References

[1] Levy D , Garrison RJ, Savage DD, Kannel WB, Castelli WP. Prognostic implications of echocardiographically determined left ventricular mass in the Framingham Heart Study. N Engl J Med 1990;322:1561–1566. 2139921 10.1056/NEJM199005313222203

[2] Devereux RB , Wachtell K, Gerdts E, Boman K, Nieminen MS, Papademetriou V, et al Prognostic significance of left ventricular mass change during treatment of hypertension. JAMA 2004;292:2350–2356. 15547162 10.1001/jama.292.19.2350

[3] Flynn JT , Kaelber DC, Baker-Smith CM, Blowey D, Carroll AE, Daniels SR, et al Clinical practice guideline for screening and management of high blood pressure in children and adolescents. Pediatrics 2017;140:e20171904. 10.1542/peds.2017-1904 28827377

[4] Flynn JT . What level of blood pressure is concerning in childhood? Circ Res 2022;130:800–808. 35239405 10.1161/CIRCRESAHA.121.319819

[5] Khoury M , Urbina EM. Cardiac and vascular target organ damage in pediatric hypertension. Front Pediatr 2018;6:148. 29881718 10.3389/fped.2018.00148 PMC5976785

[6] Agbaje AO . Elevated blood pressure and worsening cardiac damage during adolescence. J Pediatr 2023;257:113374. 36870560 10.1016/j.jpeds.2023.02.018

[7] Agbaje AO , Zachariah JP, Tuomainen TP. Arterial stiffness but not carotid intima-media thickness progression precedes premature structural and functional cardiac damage in youth: a 7-year temporal and mediation longitudinal study. Atherosclerosis 2023;380:117197. 37582328 10.1016/j.atherosclerosis.2023.117197 PMC12225735

[8] Agbaje AO. Increasing lipids with risk of worsening cardiac damage in 1595 adolescents: a 7-year longitudinal and mediation study. Atherosclerosis 2023;389:117440. 38246095 10.1016/j.atherosclerosis.2023.117440

[9] Bull FC , Al-Ansari SS, Biddle S, Borodulin K, Buman MP, Cardon G, et al World Health Organization 2020 guidelines on physical activity and sedentary behaviour. Br J Sports Med 2020;54:1451–1462. 33239350 10.1136/bjsports-2020-102955 PMC7719906

[10] DiPietro L , Al-Ansari SS, Biddle SJH, Borodulin K, Bull FC, Buman MP, et al Advancing the global physical activity agenda: recommendations for future research by the 2020 WHO physical activity and sedentary behavior guidelines development group. Int J Behav Nutr Phys Act 2020;17:143. 33239105 10.1186/s12966-020-01042-2 PMC7690200

[11] Agbaje AO , Perng W, Tuomainen TP. Effects of accelerometer-based sedentary time and physical activity on DEXA-measured fat mass in 6059 children. Nat Commun 2023;14:8232. 38086810 10.1038/s41467-023-43316-w PMC10716139

[12] Agbaje AO . Longitudinal mediating effect of fatmass and lipids on sedentary time, light PA, and MVPA with inflammation in youth. J Clin Endocrinol Metab 2023;108:3250–3259. 37310686 10.1210/clinem/dgad354 PMC10655530

[13] Agbaje AO. Associations of sedentary time and physical activity from childhood with lipids: a 13-year mediation and temporal study. J Clin Endocrinol Metab; doi: 10.1210/clinem/dgad688. Published online ahead of print 14 December 2023. PMC11180508 38097375

[14] Agbaje AO , Barker AR, Lewandowski AJ, Leeson P, Tuomainen TP. Accelerometer-based sedentary time, light physical activity, and moderate-to-vigorous physical activity from childhood with arterial stiffness and carotid IMT progression: a 13-year longitudinal study of 1339 children. Acta Physiol; doi: 10.1111/apha.14132. Published online ahead of print 21 March 2024. 38509836

[15] Agbaje AO . Mediating effect of fat mass, lean mass, blood pressure, and insulin resistance on the associations of accelerometer-based sedentary time and physical activity with arterial stiffness, carotid IMT and carotid elasticity in 1574 adolescents. J Hum Hypertens; doi:10.1038/s41371-024-00905-6. Published online ahead of print 26 February 2024. PMC11076203 38409590

[16] Guthold R , Stevens GA, Riley LM, Bull FC. Global trends in insufficient physical activity among adolescents: a pooled analysis of 298 population-based surveys with 1.6 million participants. Lancet Child Adolesc Heal 2020;4:23–35. 10.1016/S2352-4642(19)30323-2 PMC6919336 31761562

[17] van Sluijs EMF , Ekelund U, Crochemore-Silva I, Guthold R, Ha A, Lubans D, et al Physical activity behaviours in adolescence: current evidence and opportunities for intervention. Lancet (London, England) 2021;398:429–442. 34302767 10.1016/S0140-6736(21)01259-9 PMC7612669

[18] Andersson C , Lyass A, Larson MG, Spartano NL, Vita JA, Benjamin EJ, et al Physical activity measured by accelerometry and its associations with cardiac structure and vascular function in young and middle-aged adults. J Am Heart Assoc 2015;4:e001528. 25792127 10.1161/JAHA.114.001528 PMC4392434

[19] Berdy AE , Upadhya B, Ponce S, Swett K, Stacey RB, Kaplan R, et al Associations between physical activity, sedentary behaviour and left ventricular structure and function from the Echocardiographic Study of Latinos (ECHO-SOL). Open Hear 2021;8:e001647. 10.1136/openhrt-2021-001647 PMC8311330 34261776

[20] Thangada ND , Patel KV, Peden B, Agusala V, Kozlitina J, Garg S, et al Cross-sectional associations of objectively measured sedentary time, physical activity, and fitness with cardiac structure and function: findings from the Dallas Heart Study. J Am Heart Assoc 2021;10:e015601. 33615827 10.1161/JAHA.119.015601 PMC8174255

[21] Schultz MG , Park C, Fraser A, Howe LD, Jones S, Rapala A, et al Submaximal exercise blood pressure and cardiovascular structure in adolescence. Int J Cardiol 2019;275:152–157. 30509371 10.1016/j.ijcard.2018.10.060 PMC6282652

[22] Barnett TA , Kelly AS, Young DR, Perry CK, Pratt CA, Edwards NM, et al Sedentary behaviors in today’s youth: approaches to the prevention and management of childhood obesity: a scientific statement from the American Heart Association. Circulation 2018;138:e142–e159. 30354382 10.1161/CIR.0000000000000591

[23] Agbaje AO . Associations of accelerometer-based sedentary time, light physical activity and moderate-to-vigorous physical activity with resting cardiac structure and function in adolescents according to sex, fat mass, lean mass, BMI, and hypertensive status. Scand J Med Sci Sports 2023;33:1399–1411. 37035905 10.1111/sms.14365 PMC10946782

[24] Agbaje AO , Saner C, Zhang J, Henderson M, Tuomainen TP. DXA-based fat mass with the risk of worsening insulin resistance in adolescents: a 9-year temporal and mediation study. J Clin Endocrinol Metab; doi:10.1210/clinem/dgae004. Published online ahead of print 04 January 2024. PMC11319001 38173399

[25] Ross R , Janssen I, Tremblay MS. Public health importance of light intensity physical activity. J Sport Heal Sci; doi:10.1016/j.jshs.2024.01.010. Published online ahead of print 01 February 2024. PMC11282331 38307207

[26] Boyd A , Golding J, Macleod J, Lawlor DA, Fraser A, Henderson J, et al Cohort profile: the ‘children of the 90s’—the index offspring of the Avon Longitudinal Study of Parents and Children. Int J Epidemiol 2013;42:111–127. 22507743 10.1093/ije/dys064 PMC3600618

[27] Fraser A , Macdonald-Wallis C, Tilling K, Boyd A, Golding J, Davey Smith G, et al Cohort profile: the Avon Longitudinal Study of Parents and Children: ALSPAC mothers cohort. Int J Epidemiol 2013;42:97–110. 22507742 10.1093/ije/dys066 PMC3600619

[28] Northstone K , Lewcock M, Groom A, Boyd A, Macleod J, Timpson N, et al The Avon Longitudinal Study of Parents and Children (ALSPAC): an update on the enrolled sample of index children in 2019. Wellcome Open Res 2019;4:51. 31020050 10.12688/wellcomeopenres.15132.1 PMC6464058

[29] Agbaje AO , Barker AR, Tuomainen TP. Effects of arterial stiffness and carotid intima- media thickness progression on the risk of overweight/obesity and elevated blood pressure/hypertension: a cross-lagged cohort study. Hypertension 2022;79:159–169. 34784721 10.1161/HYPERTENSIONAHA.121.18449 PMC8654123

[30] Harris PA , Taylor R, Thielke R, Payne J, Gonzalez N, Conde JG. Research electronic data capture (REDCap)-A metadata-driven methodology and workflow process for providing translational research informatics support. J Biomed Inform 2009;42:377–381. 18929686 10.1016/j.jbi.2008.08.010 PMC2700030

[31] Robusto KM , Trost SG. Comparison of three generations of ActiGraph^TM^ activity monitors in children and adolescents. J Sports Sci 2012;30:1429–1435. 22857599 10.1080/02640414.2012.710761 PMC3458797

[32] Troiano RP , Berrigan D, Dodd KW, Mâsse LC, Tilert T, Mcdowell M. Physical activity in the United States measured by accelerometer. Med Sci Sports Exerc 2008;40:181–188. 18091006 10.1249/mss.0b013e31815a51b3

[33] Agbaje AO , Lloyd-Jones DM, Magnussen CG, Tuomainen TP. Cumulative dyslipidemia with arterial stiffness and carotid IMT progression in asymptomatic adolescents: a simulated intervention longitudinal study using temporal inverse allocation model. Atherosclerosis 2023;364:39–48. 36462968 10.1016/j.atherosclerosis.2022.11.011

[34] Trost SG , Loprinzi PD, Moore R, Pfeiffer KA. Comparison of accelerometer cut points for predicting activity intensity in youth. Med Sci Sports Exerc 2011;43:1360–1368. 21131873 10.1249/MSS.0b013e318206476e

[35] Migueles JH , Cadenas-Sanchez C, Ekelund U, Delisle Nyström C, Mora-Gonzalez J, Löf M, et al Accelerometer data collection and processing criteria to assess physical activity and other outcomes: a systematic review and practical considerations. Sport Med 2017;47:1821–1845. 10.1007/s40279-017-0716-0 PMC6231536 28303543

[36] Nagueh SF , Smiseth OA, Appleton CP, Byrd BF 3rd, Dokainish H, Edvardsen T, et al Recommendations for the evaluation of left ventricular diastolic function by echocardiography: an update from the American Society of Echocardiography and the European Association of Cardiovascular Imaging. J Am Soc Echocardiogr 2016;29:277–314. 27037982 10.1016/j.echo.2016.01.011

[37] Lang RM , Badano LP, Mor-Avi V, Afilalo J, Armstrong A, Ernande L, et al Recommendations for cardiac chamber quantification by echocardiography in adults: an update from the American Society of Echocardiography and the European Association of Cardiovascular Imaging. J Am Soc Echocardiogr 2015;28:1–39.e14. 25559473 10.1016/j.echo.2014.10.003

[38] Agbaje AO . Longitudinal left ventricular mass indexing for DEXA-measured lean mass and fat mass: normative reference centiles in post-pubertal adolescents and young adults. Am J Physiol - Hear Circ Physiol 2023;324:H571–H577. 10.1152/ajpheart.00045.2023 PMC10042592 36827226

[39] Timpka S , Macdonald-Wallis C, Hughes AD, Chaturvedi N, Franks PW, Lawlor DA, et al Hypertensive disorders of pregnancy and offspring cardiac structure and function in adolescence. J Am Heart Assoc 2016;5:e003906. 27799232 10.1161/JAHA.116.003906 PMC5210338

[40] Agbaje AO , Barker AR, Mitchell GF, Tuomainen TP. Effect of arterial stiffness and carotid intima-media thickness progression on the risk of dysglycemia, insulin resistance, and dyslipidaemia: a temporal causal longitudinal study. Hypertension 2022;79:667–678. 35038890 10.1161/HYPERTENSIONAHA.121.18754 PMC8823909

[41] Agbaje AO , Barker AR, Tuomainen TP. Cumulative muscle mass and blood pressure but not fat mass drives arterial stiffness and carotid intima-media thickness progression in the young population and is unrelated to vascular organ damage. Hypertens Res 2023;46:984–999. 36241708 10.1038/s41440-022-01065-1 PMC10073015

[42] de Simone G , Mancusi C, Hanssen H, Genovesi S, Lurbe E, Parati G, et al Hypertension in children and adolescents. Eur Heart J 2022;43:3290–3301. 35896123 10.1093/eurheartj/ehac328

[43] Agbaje AO . Mediating role of body composition and insulin resistance on the association of arterial stiffness with blood pressure among adolescents: the ALSPAC study. Front Cardiovasc Med 2022;9:939125. 36119740 10.3389/fcvm.2022.939125 PMC9481230

[44] Agbaje AO , Barmi S, Sansum KM, Baynard T, Barker AR, Tuomainen TP. Temporal longitudinal associations of carotid-femoral pulse wave velocity and carotid intima-media thickness with resting heart rate and inflammation in youth. J Appl Physiol 2023;134:657–666. 36727630 10.1152/japplphysiol.00701.2022 PMC10010920

[45] Agbaje AO , Barker AR, Tuomainen TP. Cardiorespiratory fitness, fat mass, and cardiometabolic health with endothelial function, arterial elasticity, and stiffness. Med Sci Sport Exerc 2022;54:141–152. 10.1249/MSS.0000000000002757 PMC8677603 34334718

[46] Rubin DB . An overview of multiple imputation. Proc Surv Res Methods Sect Am Stat Assoc 1988;16:79–84.

[47] Lorell BH , Carabello BA. Left ventricular hypertrophy. Circulation 2000;102:470–479. 10908222 10.1161/01.cir.102.4.470

[48] Golding G , Pembrey P, Jones J. ALSPAC—the Avon Longitudinal Study of Parents and Children I. Study methodology. Paediatr Perinat Epidemiol 2001;15:74–87. 11237119 10.1046/j.1365-3016.2001.00325.x

[49] Armstrong AC , Gidding S, Gjesdal O, Wu C, Bluemke DA, Lima JAC. LV mass assessed by echocardiography and CMR, cardiovascular outcomes, and medical practice. JACC Cardiovasc Imaging 2012;5:837–848. 22897998 10.1016/j.jcmg.2012.06.003 PMC3501209

[50] Mitter SS , Shah SJ, Thomas JD. A test in context: e/A and E/e′ to assess diastolic dysfunction and LV filling pressure. J Am Coll Cardiol 2017;69:1451–1464. 28302294 10.1016/j.jacc.2016.12.037

[51] Agbaje AO . Longitudinal associations of accelerometer-based sedentary time and physical activity with blood pressure progression from childhood through young adulthood: a 13-year mediation and isotemporal substitution study of 2513 children. Circulation 2023;148:A16549–A16549. 10.1161/circ.148.suppl_1.16549.

[52] Agbaje AO . Waist-circumference-to-height-ratio had better longitudinal agreement with DEXA-measured fat mass than BMI in 7237 children. Pediatr Res; doi:10.1038/s41390-024-03112-8. Published online ahead of print 05 March 2024. PMC11522001 38443520

[53] Agbaje AO . The interactive effects of sedentary time, physical activity, and fat mass on insulin resistance in the young population. J Clin Endocrinol Metab; doi: 10.1210/clinem/dgae135. Published online ahead of print 05 March 2024. PMC11651701 38441224

[54] Gaasch WH , Zile MR. Left ventricular structural remodeling in health and disease: with special emphasis on volume, mass, and geometry. J Am Coll Cardiol 2011;58:1733–1740. 21996383 10.1016/j.jacc.2011.07.022

[55] Pelliccia A , Heidbuchel H, Corrado D, Borjesson M, Sharma S. The ESC Textbook of Sports Cardiology. Online edn. ESC Publications: Oxford University Press; 2019. 10.1093/med/9780198779742.001.0001

