# Supplementary material for: Accelerometer-based sedentary time and physical activity from childhood through young adulthood with progressive cardiac changes: a 13-year longitudinal study
Source: Eur J Prev Cardiol. 2024 May 7;31(12):1480–92. doi: 10.1093/eurjpc/zwae129 (PMC11378265; doi:10.1093/eurjpc/zwae129)
Supplement: zwae129_Supplementary_Data [file zwae129_supplementary_data.pdf]

Supplementary Appendix

Accelerometer-based Sedentary Time and Physical Activity from Childhood through Young Adulthood with Progressive Cardiac Changes: A 13-Year Longitudinal Study

Andrew O. Agbaje MD, MPH, PhD[1,2]

[1]*Institute of Public Health and Clinical Nutrition, School of Medicine, Faculty of Health Sciences, University of Eastern Finland, Kuopio, Finland*

[2]*Children's Health and Exercise Research Centre, Department of Public Health and Sports Sciences, Faculty of Health and Life Sciences, University of Exeter, Exeter, UK*

Address correspondence to:
Andrew O. Agbaje, MD, MPH, PhD, FESC, FAHA, Cert. Clinical Research (*Harvard*)
Professor (associate) of Clinical Epidemiology and Child Health
Institute of Public Health and Clinical Nutrition, School of Medicine, Faculty of Health Sciences, University of Eastern Finland, Kuopio Campus.
Address: Yliopistonranta 8, P.O. Box 1627, 70211 Kuopio, Finland
E-mail: andrew.agbaje@uef.fi

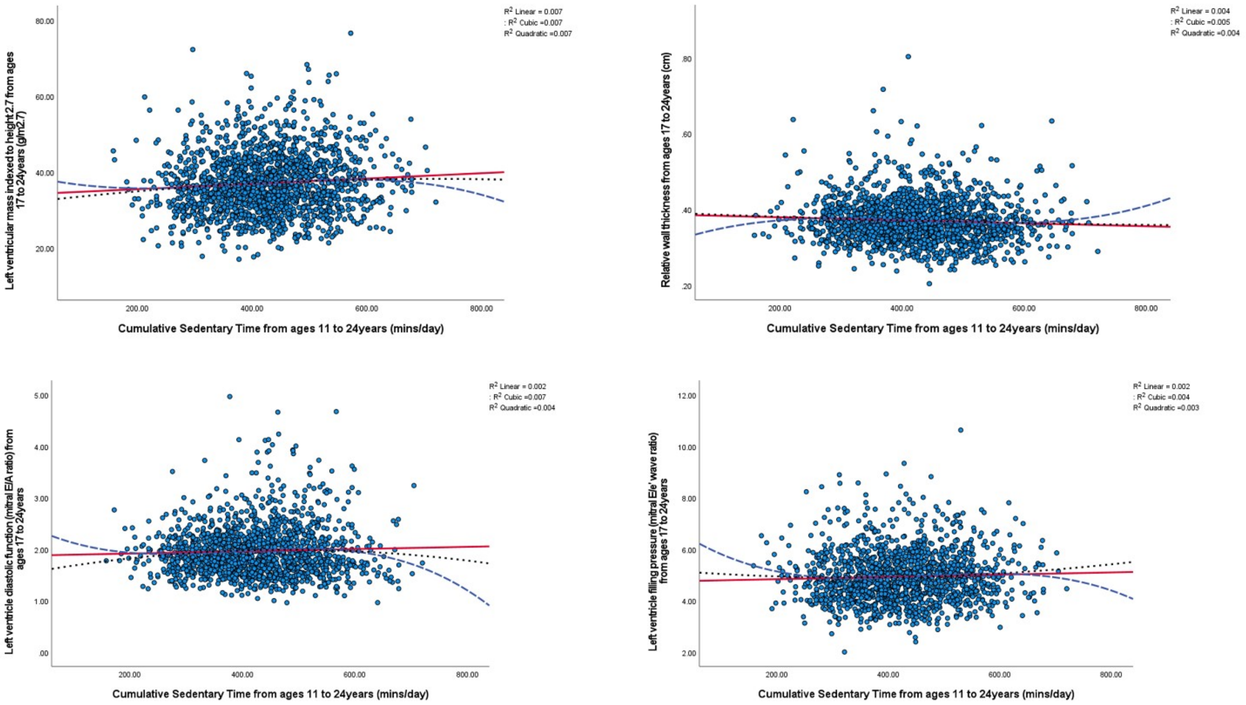

Supplemental Figure 1 Unadjusted linear, cubic, and quadratic regression plots of cumulative sedentary time (mins/day) from ages 11 – 24 years with cardiac measures from ages 17 – 24 years.

Linear regression is red solid line, quadratic regression is black dash line, cubic regression is blue long dash line.

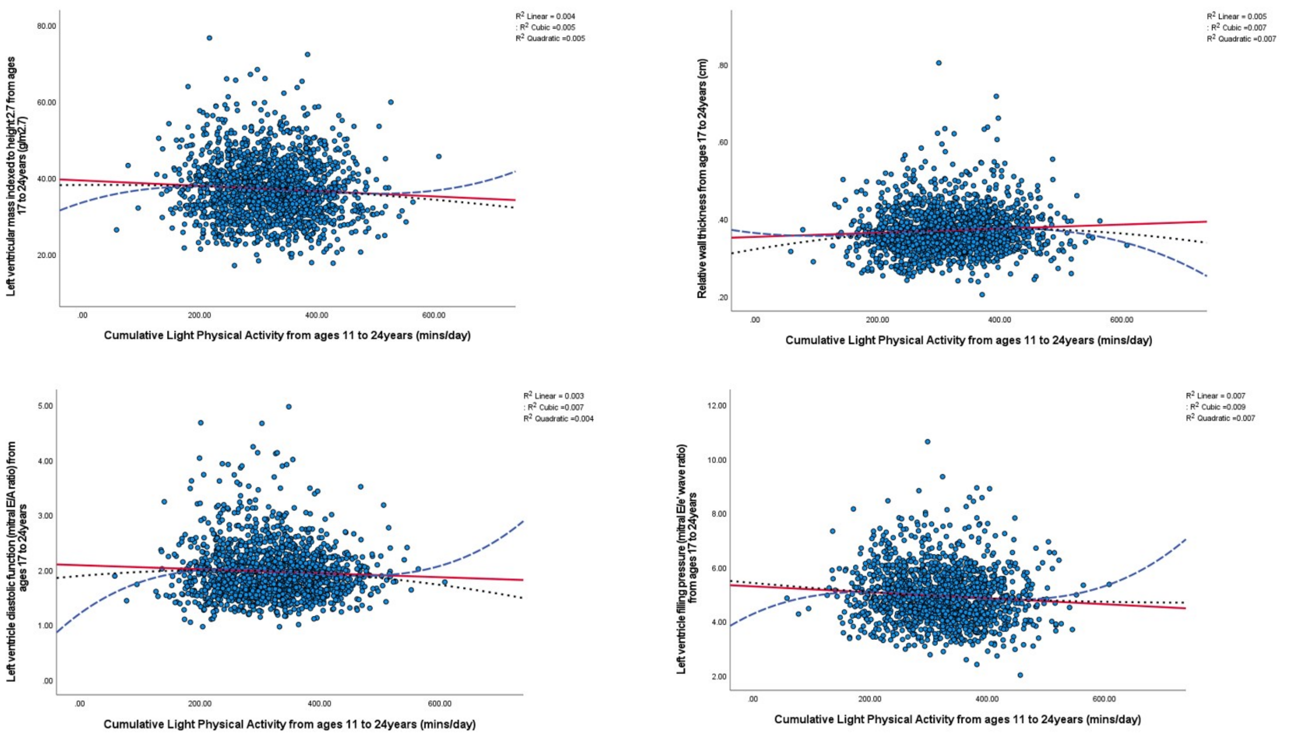

Supplemental Figure 2 Unadjusted linear, cubic, and quadratic regression plots of cumulative light physical activity (mins/day) from ages 11 – 24 years with cardiac measures from ages 17 – 24 years.

Linear regression is red solid line, quadratic regression is black dash line, cubic regression is blue long dash line. LPA, light physical activity.

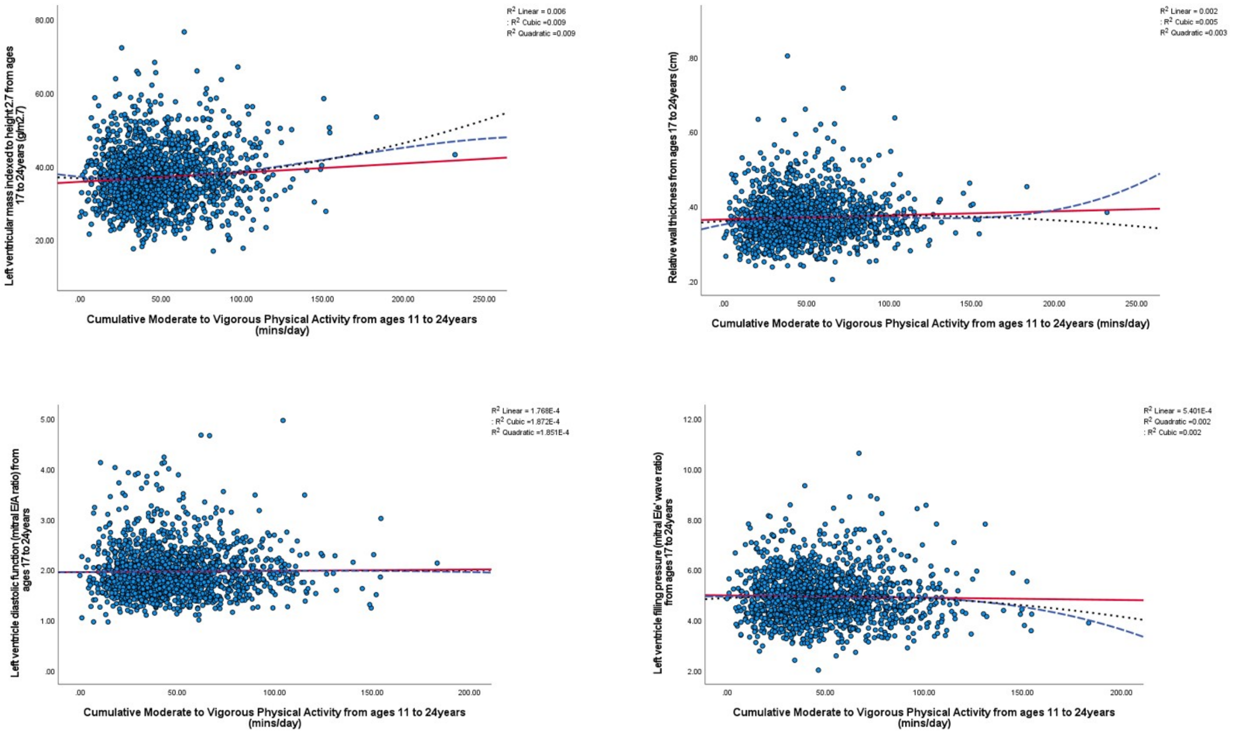

Supplemental Figure 3 Unadjusted linear, cubic, and quadratic regression plots of cumulative moderate to vigorous physical activity (mins/day) from ages 11 – 24 years with cardiac measures from ages 17 – 24 years.

Linear regression is red solid line, quadratic regression is black dash line, cubic regression is blue long dash line. MVPA, moderate to vigorous physical activity.

Supplemental Table 1 Longitudinal associations of cumulative sedentary time and physical activity from ages 11 through 24 years with the progressive change in left ventricular mass and diastolic function from ages 17 through 24 years.

N=766	$LVMI^{2.7}$ ($g/m^{2.7}$)		RWT (cm)		LVDF (E/A)		LVFP (E/e´)	
	β *(95% CI)*	*p-value*	***β*** *(95% CI)*	*p-value*	***β*** *(95% CI)*	*p-value*	***β*** *(95% CI)*	*p-value*
Continuous cumulative predictor variables from ages 11 – 24 years								
**Sedentary Time**								
Model 1	0.014 (0.012 – 0.016)	**<0.0001**	0.000 (0.000 – 0.000)	**<0.0001**	0.000 (-0.000 – 0.000)	0.602	0.004 (0.003 – 0.004)	**<0.0001**
Model 2	0.004 (0.001 – 0.006)	**0.002**	0.000 (-0.000 – 0.000)	0.646	0.000 (0.000 – -0.000)	**0.004**	0.002 (0.001 – 0.002)	**0.002**
**Light physical activity**								
Model 1	-0.019 (-0.021 – -0.017)	**<0.0001**	0.000 (0.000 – 0.000)	**<0.0001**	0.000 (0.000 – -0.000)	**<0.001**	-0.005 (-0.005 – -0.004)	**<0.0001**
Model 2	-0.017 (-0.020 – -0.015)	**<0.0001**	0.000 (0.000 – 0.000)	**<0.0001**	0.000 (0.000 – 0.000)	**<0.001**	-0.004 (-0.004 – -0.003)	**<0.0001**
**Moderate to vigorous physical activity**								
Model 1	0.003 (-0.006 – 0.011)	0.497	-0.000 (-0.000 – 0.000)	0.916	-0.001 (-0.001 – 0.000)	**0.011**	0.001 (-0.001 – 0.003)	0.207
Model 2	0.010 (0.002 – 0.019)	**0.016**	0.000 (-0.000 – 0.000)	0.207	-0.001 (-0.001 – 0.000)	**0.005**	0.003 (0.002 – 0.005)	**<0.001**
Categorical cumulative predictor variable from ages 11 – 24 years								
**Moderate to vigorous physical activity (*<40mins/day as reference)***								
40 – <60mins/day	-0.587 (-1.104 – -0.071)	**0.026**	0.007 (0.003 – 0.012)	**0.002**	-0.034 (0.065 – -0.002)	**0.036**	0.200 (0.102 – 0.297)	**<0.001**
≥60mins/day	-0.249 (-0.729 – 0.232)	0.311	0.004 (0.000 – 0.008)	0.059	-0.026 (-0.056 – 0.003)	0.081	0.343 (0.255 – 0.432)	**<0.001**

Model 1 was adjusted for sex, age at baseline and other time varying covariates measured at both baseline and follow-up such as low-density lipoprotein cholesterol, insulin, triglyceride, high-sensitivity C reactive protein, high-density lipoprotein cholesterol, heart rate, systolic blood pressure, glucose, fat mass, lean mass, smoking status, family history of hypertension/diabetes/high cholesterol/vascular disease, socioeconomic status. Model 2 was an additional adjustment for sedentary time, light physical activity or moderate to vigorous physical activity depending on the predictor. For categorical predictor variable analyses, all the above listed covariates were adjusted for in one model. Skewed covariates were logarithmically transformed. Regression coefficients ***(β)*** were computed from generalized linear mixed-effect model for repeated measures; CI, confidence interval; LVDF, left ventricular diastolic function; LVFP, left ventricular filling pressure; $LVMI^{2.7}$, left ventricular mass indexed for $height^{2.7}$; RWT, relative wall thickness, A 2-sided P-value <0.05 is considered statistically significant. Multiple testing was corrected with Sidak correction. Multiple imputations were used to account for missing variables. For continuous variable predictors (ST, LPA and MVPA), a 1-minute change is associated with the point estimate unit change in the outcome.
For categorical variable predictor (MVPA), time spent in a category in relation to the reference is associated with the point estimate unit change in the outcome.

Supplemental Table 2 Longitudinal associations of cumulative sedentary time and physical activity from ages 11 through 24 years with the progressive change in left ventricular mass and diastolic function from ages 17 through 24 years according to sex.

	LVMI$^{2.7}$(g/m$^{2.7}$)		RWT (cm)		LVDF (E/A)		LVFP (E/e´)	
	***β** (95% CI)*	*p-value*	***β** (95% CI)*	*p-value*	***β** (95% CI)*	*p-value*	***β** (95% CI)*	*p-value*
Male (n =344)								
**Sedentary Time**								
Model 1	0.032 (0.028 – 0.036)	**<0.0001**	0.000 (0.000 – 0.000)	**<0.0001**	0.001 (0.000 – 0.001)	**<0.0001**	0.004 (0.004 – 0.004)	**<0.0001**
Model 2	0.002 (-0.003 – 0.007)	0.394	0.000 (-0.0001 – 0.000)	0.512	0.000 (0.000 – 0.001)	**0.001**	0.002 (0.001 – 0.003)	**<0.0001**
**Light physical activity**								
Model 1	-0.045 (-0.048 – 0.041)	<0.0001	0.000 (0.000 – 0.000)	**<0.0001**	-0.001 (-0.001 – 0.000)	**<0.0001**	-0.004 (-0.005 – -0.004)	**<0.0001**
Model 2	-0.046 (-0.050 – -0.041)	**<0.0001**	0.000 (0.000 – 0.000)	**<0.0001**	0.000 (-0.001 – -0.000)	**0.044**	-0.003 (-0.004 – -0.002)	**<0.0001**
**Moderate to vigorous physical activity**								
Model 1	0.015 (0.002 – 0.028)	**0.022**	-0.000 (0.000 – 0.000)	0.102	0.000 (-0.001 – 0.000)	0.559	-0.002 (-0.004 – 0.000)	0.101
Model 2	0.049 (0.037 – 0.061)	**<0.0001**	0.000 (0.000 – 0.000)	**0.023**	0.000 (0.000 – 0.001)	0.168	0.003 (0.000 – 0.005)	**0.018**
Female (n=422)								
**Sedentary Time**								
Model 1	0.003 (0.000 – 0.005)	**0.020**	0.0001 (0.0001 – 0.0001)	**0.043**	0.000 (0.000 – 0.000)	**0.033**	0.000 (0.000 – 0.001)	0.318
Model 2	0.009 (0.005 – 0.012)	**<0.0001**	-0.0001 (-0.0001 – 0.0001)	0.465	-0.000 (0.000 – 0.000)	0.422	0.001 (0.000 – 0.002)	**0.004**
**Light physical activity**								
Model 1	0.002 (-0.001 – 0.004)	0.249	-0.000 (-0.000 – -0.0001)	**<0.0001**	0.000 (0.000 – 0.000)	**0.001**	0.000 (0.0001 – 0.001)	**0.035**
Model 2	0.008 (0.005 – 0.012)	**<0.0001**	-0.000 (-0.000 – -0.0001)	**0.001**	0.000 (-0.001 – -0.0001)	**0.005**	0.001 (0.001 – 0.002)	0.000
**Moderate to vigorous physical activity**								
Model 1	-0.020 (-0.032 – -0.009)	**0.001**	0.000 (0.000 – 0.000)	0.002	0.000 (-0.001 – 0.001)	0.581	-0.003 (-0.005 – 0.000)	**0.033**
Model 2	-0.013 (-0.025 – -0.001)	**0.033**	0.000 (0.000 – 0.000)	**0.002**	0.000 (-0.001 – 0.001)	0.601	-0.002 (-0.005 – 0.001)	0.164

Model 1 was adjusted for age at baseline and other time-varying covariates measured at both baseline and follow-up such as low-density lipoprotein cholesterol, insulin, triglyceride, high-sensitivity C reactive protein, high-density lipoprotein cholesterol, heart rate, systolic blood pressure, glucose, fat mass, lean mass, smoking status, family history of hypertension/diabetes/high cholesterol/vascular disease, socioeconomic status, and predictor by follow-up time interaction effect. Model 2 was an additional adjustment for sedentary time, light physical activity, or moderate to vigorous physical activity depending on the predictor. Skewed covariates were logarithmically transformed. Regression coefficients ***(β)*** were computed from generalized linear mixed-effect model for repeated measures; CI, confidence interval; LVDF, left ventricular diastolic function; LVFP, left ventricular filling pressure; LVMI$^{2.7}$, left ventricular mass indexed for height$^{2.7}$; RWT, relative wall thickness, A 2-sided P-value <0.05 is considered statistically significant. Multiple testing was corrected with Sidak correction. Multiple imputations were used to account for missing variables. For continuous variable predictors (ST, LPA and MVPA), a 1-minute change is associated with the point estimate unit change in the outcome.
